# Enhancing consumer sensory science approach through augmented virtuality

**DOI:** 10.1016/j.crfs.2024.100834

**Published:** 2024-08-31

**Authors:** Abdul Hannan Bin Zulkarnain, Howard R. Moskowitz, Zoltán Kókai, Attila Gere

**Affiliations:** a Department of Postharvest Science, Trade, Supply Chain and Sensory Science, Institute of Food Science and Technology, Hungarian University of Agriculture and Life Sciences, H-1118. Budapest, Villányi út. 29-31, Hungary; b Cognitive Behavioral Insights, LLC, Albany, NY, USA

**Keywords:** Mixed Reality (MR), Extended Reality (XR), Immersive, Consumer perception, Digital Sensory

## Abstract

Augmented Virtuality (AV) is a concept that merges components of Augmented Reality (AR) and Virtual Reality (VR), incorporating real elements into a virtual environment. This review analyses the influence of AV technology on sensory science and consumer behaviour, with the potential to improve product evaluation through sensory analysis. The objective is to develop immersive sensory environments that closely resemble real-world scenarios, offering accurate insights into consumer perceptions and preferences. Participants will be able to observe genuine food products within the virtual environment. Through the utilization of a multidisciplinary approach, the analysis explores the point at which technology and human senses intersect, revealing new and unique understandings of decision-making processes. This enhances comprehension of consumer choices and behaviour in virtual environments, providing practical uses for industries navigating the ever-changing nature of augmented virtuality. This review demonstrates that the integration of AV elements in sensory science can have a substantial influence.

## Highlights

• AV has significant potential in sensory science.
• AV has the ability to change the environment but is able to see the food in the virtual environment.
• Tracking devices can be combined to gain greater insight into consumers' perceptions and preferences.

## Graphical abstract

**Figure:**
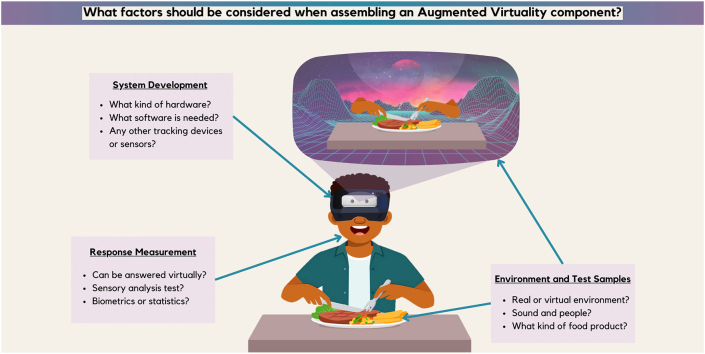

## 1 Introduction

The concept of augmented virtuality (AV) has not yet received significant attention in diverse research domains, especially in the fields of sensory and consumer science. AV combines components of virtual reality (VR) and augmented reality (AR) with tangible elements or actual products, resulting in a distinctive and immersive encounter. Augmented and virtual reality technologies have been widely employed to replicate real-life experiences and explore how consumers perceive and interact with food products (also referred as object) (Gere et al., 2021a). However, AV takes a unique approach by incorporating actual objects from the real world into virtual environments.

**Fig. 1:**
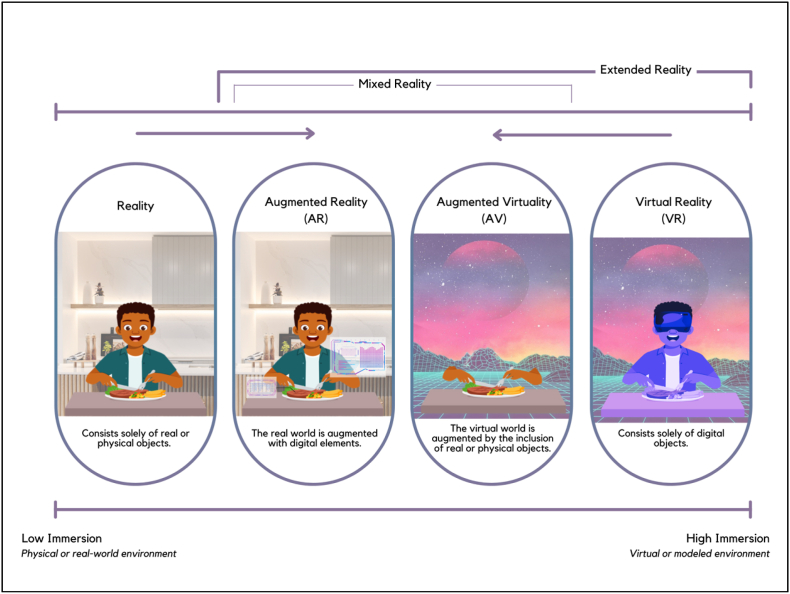
Illustration of Reality-Virtuality Continuum in a concept of sensory and consumer science inspired by Milgram and Kishino (Milgram and Kishino, 1993).

Fig. 1 shows the reality-virtuality continuum, as initially suggested by Milgram and Kishino (1993). This continuum functions as a conceptual framework for comprehending the range of possibilities between the tangible, actual world and completely virtual environments. AV is a concept that combines virtual environments with real-world elements (Low et al., 2024), effectively bridging the gap between reality and virtuality.

VR typically provides users with a fully artificial environment, while AR adds digital content to the real world. On the other hand, AV seeks to integrate real elements or products into a virtual environment. AR and AV are components of mixed reality (MR), which is characterized by the integration of real and virtual elements (Qamar et al., 2023). However, MR stands out because it effortlessly combines the physical and digital realms, enabling instantaneous interaction between tangible and virtual entities. Unlike AR, which enhances the real world by adding digital overlays, or AV, which incorporates real-world elements such as food products into a virtual environment, MR creates a completely immersive experience where physical and virtual objects coexist and interact. AV combines real-world and virtual elements to create a distinctive and immersive experience that maximizes the advantages of both. AV fundamentally entails the incorporation of tangible objects, information, or interactions from the physical world into virtual environments, thereby enhancing the user's overall experience.

An aspect of AV involves integrating tangible objects from the physical world into virtual environments. This may involve the depiction of physical entities, such as tools, machinery, or consumable goods utilized in particular sectors, within computer-generated simulations. For example, in the field of food sensory science, AV can be employed in various ways to create more realistic contexts for examining consumer perceptions, preferences, and behaviours towards food products. Scientists can create computer-generated simulations of food environments where participants can interact with actual food items and experience various sensory characteristics like taste, smell, texture, and appearance. This setup aims to generate information that is more ecologically valid by reflecting real-world conditions. Participants provide feedback and make observations in a simulated setting, enabling an organized study of how sensory stimuli impact consumer reactions and the cognitive processes involved in decision-making. This approach helps in obtaining more realistic insights into consumer behaviour.

## 2 Augmented virtuality concept in food sensory science and gap identification

One of the primary advantages of utilizing augmented virtuality (AV) in food sensory science is its capacity to generate controlled and repeatable sensory experiences for research and testing purposes. By employing AV technology, researchers can manipulate virtual environments within real food products to investigate consumer preferences, behaviours, and reactions under precisely controlled conditions. This controlled setting allows for the isolation of specific variables, enabling researchers to study the impact of individual factors on sensory perceptions without external influences. Traditional sensory testing methods often face challenges related to controlling external variables such as environmental conditions, presentation variations, and human biases, which can introduce inconsistencies and inaccuracies in the data collected. In contrast, AV technology provides researchers with a controlled virtual environment where they can standardize stimuli presentation, manipulate sensory attributes, and systematically vary factors of interest to observe their effects on consumer responses. However, one critical limitation of AV is its reliance on advanced technology and equipment, which can be costly and may limit its accessibility for smaller research institutions.

**Fig. 2:**
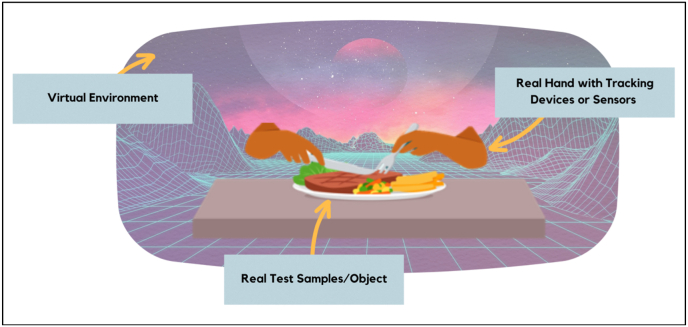
Concept of Augmented Virtuality blending the virtual environment with real test samples or products.

Another important aspect of AV is measuring the level of consumer presence and engagement. Adapting AV to virtual reality (VR) and augmented reality (AR) studies have shown that AV can offer a more seamless integration of real and virtual elements, potentially leading to higher levels of presence and engagement. For instance, the ability of AV to incorporate real food products into a virtual environment can create a more immersive and realistic experience compared to VR, which relies entirely on virtual stimuli, or AR, which overlays digital information in the real world without fully integrating the two (Fig. 2).

Recent studies have shown a growing interest in the use of immersive technologies to study consumer behaviour, sensory perception, and food experiences in the food industry and academia (Bhavadharini et al., 2023). While there is research on the immediate effects of these technologies on sensory perception and emotional responses to food products (Fuentes et al., 2021), there remains a gap in understanding the long-term impact of augmented virtuality on consumer decision-making and behaviour towards food and beverage consumption. Focusing solely on AV, it is essential to assess how AV specifically influences consumer preferences, purchasing behaviours, and product acceptance over time. Despite the potential benefits, AV technology also presents challenges such as ensuring the realism and accuracy of virtual elements integrated with real-world products, which can affect the validity of sensory data collected.

In addition, certain products may be more suited to AV over VR or AR. Products that require a high level of sensory interaction, such as foods that involve complex textures or aromas, can benefit significantly from the capabilities of AV. The integration of real sensory attributes with virtual enhancements allows for a more accurate and comprehensive evaluation of such products. In contrast, simpler products that do not require as much sensory detail might not derive as much benefit from the additional complexity of AV compared to VR or AR.

Some concepts that can be adapted from related immersive technologies into AV include enhancing consumer attraction to food products through visually captivating experiences and conveying environmental and nutritional information effectively. For instance, integrating virtual objects with the real world to create immersive experiences can influence various aspects of consumer behaviour (Fritz et al., 2023; Sonderegger et al., 2019; Mellos and Probst, 2022) or AR product presentation can improve consumer evaluation, boosting food retailer sales, repurchase intentions, and word-of-mouth (Jeganathan and Szymkowiak, 2023). This adaptation could further enhance the practical applications of AV in sensory science and consumer behaviour research. Nevertheless, the critical challenge remains in seamlessly integrating these elements without compromising the natural sensory attributes of real food products.

Similarly, insights from mixed reality (MR) and VR can inform AV applications. MR systems' ability to influence meal experiences and social interactions can be leveraged in AV to create more engaging and pleasant sensory environments (Korsgaard et al., 2020). Fuchs et al. (2020) suggest MR headsets for diet tracking and interventions, showing their feasibility in promoting healthier food choices in real time. Additionally, MR can evoke similar emotional responses in real-life settings, offering a promising tool for assessing consumer reactions accurately (Low et al., 2021). This body of research highlights MR's versatility in enhancing understanding of consumer behaviour and sensory experiences in various food-related contexts, which can be brought into the concept of AV. However, AV must address specific technical challenges such as latency and synchronization issues between real and virtual elements to maintain a coherent user experience.

VR is indeed a promising tool in sensory science and consumer behaviour research, providing immersive experiences that influence perceptions and choices. It shares a close relationship with AV concepts, where some studies in VR can serve as advantages in AV applications. Harris et al. (2023) showed VR's effectiveness in eliciting real cravings through immersive food stimuli, indicating its potential to study food effects on behaviour. Wan et al. (2022) used VR to study colour contrast and sustainable food choices, highlighting its ability to simulate real-world scenarios. Arrazat et al. (2023) studied food choices in a virtual supermarket, demonstrating VR's potential for realistic shopping environments. Zulkarnain et al. (Zulkarnain et al., 2024; Zulkarnain et al., 2024a) introduced a virtual sensory laboratory for evaluations, showcasing VR's potential to revolutionize sensory analysis practices. These studies underscore VR's versatility in advancing sensory science and understanding consumer behaviour in food perception and choice. Collectively, these studies demonstrate the versatility and promise of VR in advancing sensory science and consumer behaviour research within the realm of food perception and choice, and the research in VR has demonstrated its effectiveness in eliciting real cravings and studying food effects on behaviour, which could be valuable in designing AV environments that integrate real-world sensory stimuli. By leveraging insights and methodologies from VR research, AV applications can enhance immersion and realism, ultimately improving the understanding of consumer behaviour and sensory experiences. Nonetheless, the challenge for AV remains in ensuring that the integration of virtual and real elements does not distort the sensory experiences intended for assessment. Table 1 shown the examples of research conducted on sensory analysis using one or more types of realities [Augmented Reality (AR), Mixed Reality (MR) and Virtual Reality (VR)] and their main findings. More details and the possible adaptation of AV in AR, MR and VR studies are listed in Table S1.

**Table 1:** Research conducted on sensory analysis using one or more type(s) of reality [Augmented Reality (AR), Mixed Reality (MR) and Virtual Reality (VR)] and their main findings.

Reference	Types(s) of Reality	Main Findings
(Jeganathan and Szymkowiak, 2023)	AR	The study found that environmental embedding and product liking are mediated by mental imagery quality, affecting the indirect relationship between the two.
(Low et al., 2021)	MR	Researchers found that consumers can be categorised by their emotional response to tea-break snacks, with one emotionally disengaged group and two positively engaged groups at varying intensity and qualities.
(Fritz et al., 2023)	AR	Research indicates that AR display enhances food item attractiveness and purchase likelihood by boosting mental simulation and personal relevance.
(Mishra et al., 2021)	AR	The study found that multimodal technologies, particularly AR, increase visual appeal, emotional appeal, and purchase intentions for hedonic products over utilitarian products.
(Korsgaard et al., 2020)	MR	The main finding of the study was that energy intake did not change, but the virtual living room environment was perceived to be more energetic and pleasant, and meals eaten there were rated as higher quality than those eaten in a lab.
(Mellos and Probst, 2022)	AR	The research found that nutrition students' food portion estimate skills may improve with AR tools.
(Fuchs et al., 2020)	MR	The study found that MR headset-mediated visual treatments positively impacted beverage and food choices, reducing sugar, energy intake, and saturated fat intake and increasing healthy product selection.
(Torrico, 2021)	VR	The study paper focuses on developing new methodologies to quantify customers' sensory, emotional, and physiological responses to food products.
(Crofton et al., 2021)	VR	The study found that particular contexts, like a VR restaurant for beef and a countryside for chocolate, significantly affect participants' sensory responses to food products compared to traditional lab settings.
(Seo, 2020)	VR	Environmental cues around foods and beverages can modulate consumer perception, emotional responses, and behaviour, highlighting the importance of sensory cues from surrounding contexts in understanding food product consumer behaviour.
(Bhavadharini et al., 2023)	AR, MR, VR	The research paper uses immersive technologies like virtual, augmented, and mixed reality to evaluate food consumer behaviour, focusing on product selection, shopping behaviour, and emotional influence on product choices.
(Strecker et al., 2024)	MR	ShoppingCoach, a Diminished Reality (DR) prototype, improves users' compliance with dietary recommendations and speeds up product selection, suggesting that DR could help people eat healthier.
(Gere et al., 2021a)	VR	The paper introduces current VR knowledge in food science and highlights the most important questions about VR applications in this field.
(Zulkarnain et al., 2024)	VR	The study found that combining VR technology with scent identification techniques provides a novel approach to sensory analysis, influencing scent perception and exposure through imagery or the environment.
(Zulkarnain et al., 2024b)	VR	The research found that the VR-enabled virtual sensory booth could improve sensory evaluation and perception studies, particularly in sensory science.
(Zulkarnain et al., 2024a)	VR	The main finding of the study was the development of a virtual sensory laboratory with a focus on sensory perception in a virtual environment.
(Fuentes et al., 2021)	AR,MR,VR	The research paper highlights the potential for digital technologies to reduce biases and subjectivity in sensory science, incorporating physiological and emotional responses of panellists for accurate data acquisition and interpretation, potentially revolutionizing the food and beverage industries.
(Lombart et al., 2019)	VR	The study found that consumers prefer ‘moderately’ misshapen fruits and vegetables due to their appearance and quality, compared to ‘slightly’ and ‘heavily’ misshapen ones.
(Schouteten et al., 2024)	VR	The study found that food products were more liked when consumed in a congruent VR context, with no difference in elicited emotions.
(Yang et al., 2022)	VR	The study found that the VR session had higher participant engagement than the Evoked session, with audio, VR time, and realistic simulations enhancing immersion.
(Ammann et al., 2020)	VR	The study concluded that sensory studies can be successfully transferred to VR environments, resulting in comparable results to real-life settings.
(Chai et al., 2022)	AR,MR	The research reveals that AR/MR technology is primarily used in dietary assessment, food nutrition and traceability, sensory science, retail food chain applications, food education and learning, and precision farming. However, limitations and analytical challenges hinder its use in the food sector.
(Van Der Laan et al., 2022)	VR	The study found no significant preference for perceived healthy or unhealthy food products across prime conditions.
(Zhang et al., 2022)	VR	The study found that presenting a meat dish in a red container and a vegetable dish in a blue container led to more vegetable dish choices and fewer meat dish choices, highlighting the impact of container colour on food choices.
(Torrico et al., 2021)	VR	The study found that virtual reality (VR) environments did not significantly impact the liking of full-sugar and no-sugar chocolate attributes, but did impact the intensity of sweetness and emotional responses to the products.
(Torrico et al., 2020)	VR	The main finding of the study was that the context (booths, real, or VR) significantly influenced the perception of the wine's floral aroma, while the liking of sensory attributes remained consistent across different environmental conditions.

**Fig. 3:**
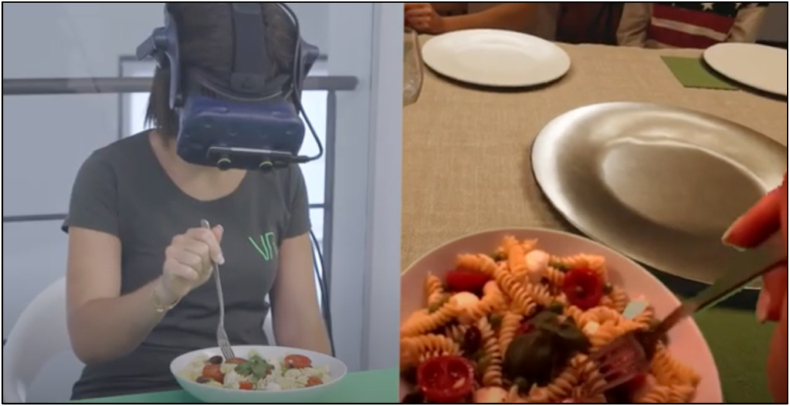
A clip of a video on the potential of AV in a sensory analysis titled “Contextualized Tasting Experience in Augmented Virtuality” (https://www.youtube.com/watch?v=ZkBQbDQiRUg&ab_channel=INVRSION) created by Invrsion S.r.l. (Milan, Italy) VR software company.

The studies have highlighted the potential impact of immersive technologies on creating immersive and interactive experiences that evoke a sense of presence in virtual environments. This can ultimately influence consumer empathy and behavioural intentions (Chai et al., 2022). However, there is a lack of research examining the specifics of AV experiences and their impact on consumer responses. To address this gap, we can adapt findings from related studies to identify potential moderating factors that influence the effectiveness of AV interventions in shaping consumer attitudes and behaviours. For example, Fig. 3 shows a part of a video clip from a VR software company called Invrsion S.r.l. (Milan, Italy). The video showcases the potential of AV technology in sensory analysis studies. However, significant research is needed to optimize AV technology for sensory evaluations, particularly in ensuring that virtual enhancements do not overshadow or misrepresent the real food products being tested.

## 3 Augmented virtuality key component overview

**Fig. 4:**
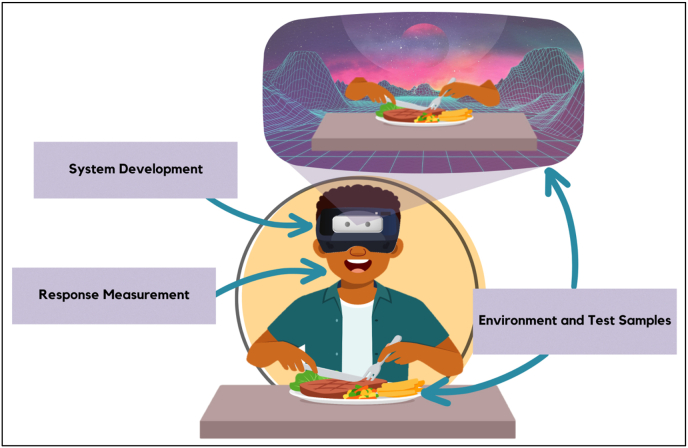
Augmented Virtuality key components that should be considered.

Creating an augmented virtuality (AV) study involves looking at its key parts (Fig. 4), which are System Development, Response Measurement, and Environment and Test Samples. These parts are like building blocks that help researchers get a better understanding of the components needed as shown in the concept in Fig. 2, Fig. 3.

Zulkarnain et al. (2024b) have developed an application on Virtual Reality (VR) that can potentially transformed into an AV application. They suggest that VR Sensory booths can be utilized to create immersive experiences by incorporating different sensory methods in various environments. The place of measurement AV can easily be an empty table with a white or green background, so it masks the test samples or objects in the virtual environment. This approach can significantly enhance the development of AV applications, paving the way for more realistic and engaging user experiences.

### 3.1 System development

**Fig. 5:**
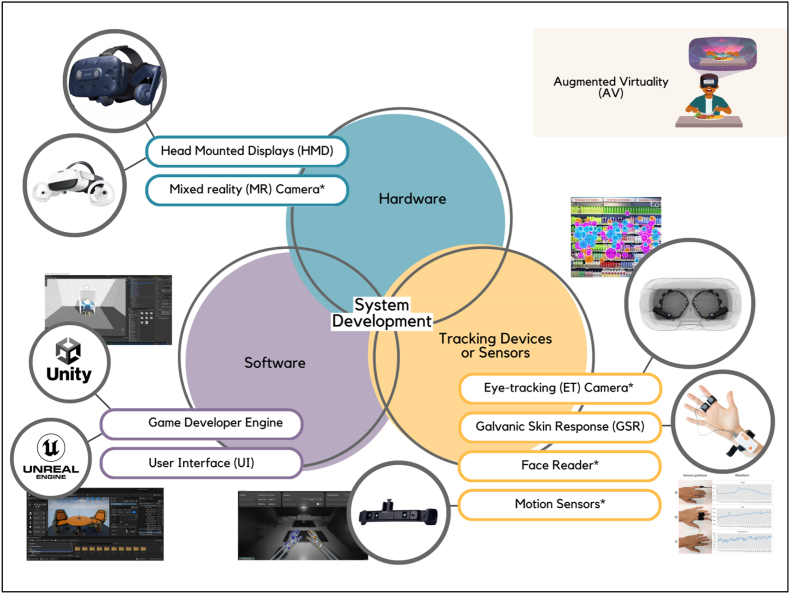
The system development on hardware components, software applications and integrating tracking devices or sensors with some examples of picture or graphical representation. (*Not necessary to have as they are built into some of the Head Mounted Displays).

In the comprehensive system development of AV, a various approach is adopted with some examples of picture or graphical representation in Fig. 5, intertwining hardware components, and software applications, and integrating tracking devices or sensors into the framework to enable precise spatial mapping and interaction, thereby constructing a seamless and immersive virtual environment for users to engage with.

Head Mounted Displays (HMDs) are wearable devices resembling glasses that are utilized in Virtual Reality (VR) and Augmented Reality (AR) applications. These devices offer immersive experiences by displaying virtual content or introducing additional elements into the real world (Ukai et al., 2021). Examples include HTC Corporation (Xindian, New Taipei, Taiwan), Meta Platforms Technologies (Menlo Park, California, U.S.), and Pico Immersive Pte. Ltd. (Tokyo, Japan). Head-mounted displays (HMDs) are highly suitable for Augmented Virtuality (AV) as they enable users to perceive and engage with virtual and real elements concurrently. When conducting AV research that emphasizes sensory experiences with actual products, the main objective is typically not to sustain an uninterrupted perception of the physical environment. Instead, the focus is on controlling or enhancing the perception of the actual product within a virtual environment. Immersive virtual reality head-mounted displays (HMDs) can effectively accomplish this by providing precise manipulation of both auditory and visual stimuli. This capability allows researchers to isolate and study specific sensory elements of the product. While, Mixed Reality (MR) cameras are also crucial in AV. They blend real-life scenes with computer-generated images, making augmented reality more realistic (Khatib et al., 2021). Examples include Stereolabs Inc. (San Francisco, U.S.) and Intel RealSense Technology (Santa Clara, California, U.S.). However, nowadays, most HMDs come with MR cameras already integrated.

Software, especially game development engines, is crucial in making AV experiences. These tools help make video games by coding, designing graphics, adding sound, and managing game parts (Chia et al., 2020). Unreal Engine, developed by Epic Games, Inc. (Cary, North Carolina, U.S.), and Unity from Unity Technologies (San Francisco, California, U.S.) are some examples of software for VR. These engines have strengths and weaknesses for AV development. Based on the studies (Zulkarnain et al., 2024a, 2024b), Unreal Engine excels in delivering photorealistic graphics and immersive experiences, making it ideal for projects that require high visual fidelity and realism. However, its steep learning curve may pose challenges for beginners. Unity, on the other hand, prioritizes accessibility and rapid development, making it a popular choice for indie developers and small teams. Its user-friendly interface and extensive documentation make it easier for newcomers to get started with AV development. While Unity may not offer the same level of graphical fidelity as Unreal Engine out of the box, its flexibility and ease of use make it a compelling choice for projects where time-to-market and iteration speed are crucial factors.

Tracking Devices or Sensors are tools used to gather data about how people interact with things in AV. Eye-tracking cameras follow where people look at, helping understand how people choose food or products (Gere et al., 2021b). Examples include Tobii AB (Danderyd Municipality, Sweden) and iMotions A/S (Copenhagen, Denmark). Another sensor are Galvanic Skin Response (GSR) sensors which measure electrical changes in skin to understand emotions or reactions to food (Tonacci et al., 2019), such as, Shimmer Research Ltd (Dublin, Ireland), Maxim Integrated Products Inc (San Jose, California, U.S.), and Mindfield Biosystems Ltd (Gronau, Germany). Face readers interpret facial expressions to discern emotions or traits, utilizing facial expression analysis technology such as facial electromyograms and facial gesture recognition, to enable hands-free user interfaces and immersive social interactions within virtual reality environments (Cha and Im, 2022). Most HMDs have a built-in face reader that tracks lip movement. Last but not least, motion sensors, track the movements of the user's body and head to translate them into corresponding actions within the virtual environment, enhancing immersion and interaction (Liliana et al., 2020). Examples include Magic Leap, Inc (Plantation, Florida, U.S.) and most HMDs also have a built-in front camera that tracks hand gestures. Tracking Devices or Sensors are essential components of AV development, enabling developers to gather valuable data on user interactions, physiological responses, and expressions. By leveraging this data, developers can create more engaging, personalized, and immersive AV experiences that effectively respond to user actions and emotions.

### 3.2 Response Measurement

Response Measurement, especially in food sensory analysis using AV technology, is crucial for advancing AV experiences. By accurately capturing and analysing participants' responses to virtual food stimuli, developers can improve the realism, effectiveness, and reliability of AV applications in food research and product development. AV technology transforms traditional evaluation methods by allowing virtual elicitation of responses from participants. Response Measurement is essential for enhancing the depth, accuracy, and reliability of sensory evaluation in AV development for food research and product development. By effectively capturing and analysing participants' responses, developers can create more immersive, engaging, and personalized AV experiences that drive innovation in the food industry and offer tailored sensory experiences to consumers.

Using VR headsets or immersive displays, individuals can interact with virtual food items, providing feedback on taste, aroma, texture, and appearance (Gagaoua et al., 2022). This method breaks geographical barriers, enabling data collection from diverse populations under controlled experimental conditions. Biometric technology, like measuring autonomic nervous system reactions, captures subconscious sensory and emotional responses to food stimuli, offering reliable assessments beyond conscious control (Biju et al., 2021).

Moreover, AV can revolutionize sensory analysis tests such as preference tests, triangle tests, just-about-right, check-all-that-apply (Ares and Jaeger, 2015), or rate-all-that-apply methods (Ares et al., 2014) by simulating real-world environments. Through interactive virtual platforms, participants can evaluate food attributes authentically, enhancing engagement and flexibility in experimental design (Zulkarnain et al., 2024b). Statistical analysis techniques, like multivariate analysis methods, help uncover patterns and correlations in participants' responses, providing a comprehensive understanding of sensory perception and interaction with virtual food stimuli (Crofton et al., 2019).

Motion sickness and system development questionnaires in AV can be essential for understanding issues like simulator sickness, system faulty and the environment. Some examples of questionnaires like SSQ (Simulator Sickness Questionnaire) (Kennedy et al., 1993), ARSQ (Augmented Reality Sickness Questionnaire) (Hussain et al., 2021), VRNQ (Virtual Reality Neuroscience Questionnaire) (Kourtesis et al., 2019), and PANAS (Positive and Negative Affect Schedule) (Watson et al., 1988) measure participants' comfort levels and overall experiences. These tools help assess how comfortable people feel when using AV technology, which is crucial for ensuring a positive user experience. By collecting responses through these questionnaires, researchers can identify factors that contribute to discomfort and make improvements to enhance comfort and usability. Studies on VR have shown that comfort plays a significant role in how people perceive and interact with virtual environments (Zulkarnain et al., 2024a, 2024b, 2024c). By measuring responses through questionnaires like SSQ, ARSQ, VRNQ, and PANAS, researchers can gain valuable insights into the importance of comfortability in AV experiences. This understanding can guide the development of more user-friendly and enjoyable AV applications in the future.

Overall, AV technology, combined with biometric and statistical analysis, enhances the depth and accuracy of sensory evaluation in food research, leading to innovative product development and personalized sensory experiences.

### 3.3 Environment and Test Samples

In exploring augmented virtuality (AV) for food sensory evaluation, both real and virtual environments are crucial. Real settings provide tangible sensory cues like texture, aroma, and appearance, adding authenticity to food assessment. Virtual environments offer flexibility and control, allowing researchers to simulate different scenarios and manipulate sensory factors. Combining both realms optimizes sensory testing by blending the realism of physical settings with the adaptability of virtual simulations. Since AR technologies have been used in gastronomy to compare visual expectations of real and virtual food products (Çöl et al., 2023), it is also can be used in AV.

Sound and real products (refer as object) are key in AV food sensory evaluation. Sound influences perception, affecting taste, texture, and overall sensory experience. Adding ambient sounds from real or simulated environments enriches sensory testing, enhancing immersion and authenticity. Objects contribute to the touch and the feel of presence aspects of food evaluation, influencing perceptions through interactions and sight. Integrating these elements in AV environments helps capture comprehensive sensory assessments, reflecting diverse responses and preferences (Wang et al., 2021). Additionally, nanowire-based soft wearable interfaces have been developed to enhance the sensory experience in virtual and augmented reality applications (Wang et al., 2021).

Regarding food products, AV sensory testing covers a range from beverages and snacks to complex dishes. Each product has unique sensory traits, allowing researchers to explore taste, aroma, appearance, and texture. By selecting various food items, researchers can assess the versatility of AV technology across culinary experiences, enriching sensory science and consumer insights. Virtual and augmented reality technologies show potential in sensory science, especially in studying meal choices and testing usability in a virtual reality food court (Chai et al., 2022; Gere et al., 2021b). Moreover, these technologies relate to consumer consciousness in multisensory extended reality, emphasizing their impact on perception and psychology (Petit et al., 2022).

In AV food sensory evaluation, test samples span various food products, from beverages and snacks to complex dishes. Each product offers distinct sensory features, enabling researchers to delve into taste, aroma, appearance, and texture. By including a diverse array of test samples, researchers can gauge the versatility of AV technology across culinary experiences, enhancing sensory science and consumer understanding.

## 4 Limitations and consideration of augmented virtuality development

The use of augmented virtuality (AV) in food sensory science presents several limitations, particularly concerning lighting, display resolution, ethical concerns, and user experience. When considering the visual aspects of food perception, the quality of lighting and display resolution in AV environments can significantly impact the accuracy and realism of the sensory experience. This limitation has been addressed within augmented reality (AR), mixed reality (MR), and virtual reality (VR) developments, which should be considered when developing AV.

One limitation pertains to the challenge of replicating natural lighting conditions within virtual environments (Mostafavi et al., 2024). The ability to accurately simulate natural lighting, including variations in intensity, colour temperature, and direction, is crucial for creating realistic food appearances in AV. Inadequate lighting simulation may lead to discrepancies in colour perception, texture rendering, and glossiness of food items, potentially influencing participants' sensory evaluations and affecting the reliability of research outcomes.

Furthermore, the resolution display in AV systems can pose limitations in representing fine details and textures of virtual food products (Zulkarnain et al., 2024a; Ammann et al., 2020). Insufficient display resolution may result in pixelation, blurriness, or loss of intricate visual attributes, which are essential for conveying the realistic appearance of food items. This limitation can compromise the fidelity of the virtual sensory experience, potentially impacting participants' ability to discern subtle visual cues and leading to less accurate assessments of food attributes.

Ethical considerations are very important in AV development, particularly regarding participant well-being and data privacy. Researchers must ensure informed consent, minimize potential discomfort or disorientation caused by immersive experiences, and safeguard participants' personal information collected during sensory evaluations (Rudschies and Schneider, 2024). User experience is also a critical aspect to consider in AV design. User interfaces should be intuitive, accessible, and conducive to meaningful interaction with virtual food stimuli. Engaging and enjoyable experiences contribute to the effectiveness and reliability of sensory evaluations conducted in AV environments.

Moreover, the technological constraints and high costs associated with AV are significant limitations for both researchers and industry. Advanced AV setups often require sophisticated hardware and software, which can be prohibitively expensive (Bekele and Champion, 2019). However, it's worth noting that many head-mounted displays (HMDs) are becoming more affordable, making the technology increasingly accessible. Despite this, the overall cost of comprehensive AV systems, including software development and maintenance, remains high and can limit widespread adoption.

These limitations highlight the importance of addressing the technical challenges associated with AV development. Researchers and developers must work together to improve lighting simulation, enhance display resolution, uphold ethical standards, and prioritize user experience to maximize the potential of AV in food sensory research. Additionally, interdisciplinary collaborations between sensory scientists, computer graphics experts, and display technology specialists are essential to overcome these limitations and advance the application of AV in food sensory research.

## 5 Future applications

The future application of Augmented Virtuality (AV) in food sensory science holds significant promise for revolutionizing sensory research, consumer engagement, and product development within the food industry. As technology continues to advance, the integration of AV in food sensory science is poised to offer a multitude of innovative applications and benefits.

One of the future applications of AV in food sensory science lies in the domain of personalized sensory experiences. With the advancement of AV technology, researchers and food industry professionals can tailor virtual sensory environments to individual preferences, allowing for personalized product evaluations and consumer interactions. This personalized approach has the potential to enhance consumer satisfaction and facilitate the development of customized food products that cater to specific sensory preferences and dietary needs.

The innovative technology of AV offers unprecedented opportunities in the field of food sensory science. By seamlessly integrating real food products into virtual sensory experiences, AV has the power to revolutionize food research, product development, and consumer engagement. One of its most significant applications is the creation of a virtual environment where users can taste and experience real food products while being immersed in a virtual world (Zulkarnain et al., 2024a; Crofton et al., 2021; Schouteten et al., 2024). Unlike VR, AV allows for real food to be visible in the virtual environment, enabling rapid prototyping and flavour profile testing without the need for physical travel.

In consumer research, AV has the potential to transform traditional methods by facilitating virtual tasting sessions (Zulkarnain et al., 2024b). Participants can explore a vast array of food products in customizable environments, providing real-time data on taste preferences and emotional responses, which can inform product optimization strategies. Additionally, AV allows for remote participation in sensory evaluations, promoting inclusivity and expanding the reach of sensory research initiatives.

Looking ahead, AV could also be used to develop interactive educational tools for sensory training and consumer education. AV simulations could provide realistic scenarios for training sensory professionals, such as food scientists, chefs, and sommeliers, allowing them to refine their sensory evaluation skills in a virtual environment. Moreover, AV experiences could be designed to educate consumers about the sensory characteristics of different foods and beverages, promoting greater awareness and appreciation of diverse culinary traditions and flavours. AV in food sensory science has the potential to transform the way we perceive, interact with, and consume food, driving innovation and enhancing the consumer experience.

## 6 Conclusion

In conclusion, the integration of Augmented Virtuality (AV) in sensory science has the potential to bring about a paradigm shift in our understanding of human perception, behaviour, and preferences in relation to food. AV technology enables researchers to explore sensory evaluation in unique and innovative ways, surpassing the limitations of traditional methods. The blending of virtual and real-world elements in AV offers unprecedented opportunities to study the complex interplay between sensory stimuli, environmental factors, and individual responses.

The impact of AV in sensory science is not limited to the laboratory. It has implications for diverse fields such as food product development, marketing, and consumer engagement. By providing insights into consumer preferences, AV empowers industry stakeholders to innovate and customize products to meet evolving consumer demands. Furthermore, AV deepens our understanding of cultural, social, and psychological influences on sensory perception, enriching our knowledge of human behaviour in the context of food consumption.

As AV technology is evolving, its potential to revolutionize sensory science is limitless. However, it is essential to address technical challenges, ethical considerations, and user experience optimization through ongoing collaboration between researchers, technologists, and industry partners. By using AV in a responsible and ethical manner, we can unlock new frontiers in sensory research, leading to more informed decision-making, enhanced consumer experiences, and ultimately, a healthier and more sustainable food future.

## CRediT authorship contribution statement

**Abdul Hannan Bin Zulkarnain:** Conceptualization, Methodology, Software, Formal analysis, Investigation, Writing – original draft, Writing – review & editing, Visualization. **Howard R. Moskowitz:** Writing – review & editing. **Zoltán Kókai:** Supervision, Writing – review & editing. **Attila Gere:** Conceptualization, Methodology, Validation, Writing – original draft, Writing – review & editing, Supervision.

## Declaration of competing interest

The authors declare that they have no known competing financial interests or personal relationships that could have appeared to influence the work reported in this paper.

## Data Availability

No data was used for the research described in the article.
