# Supplementary material for: Enhancing consumer sensory science approach through augmented virtuality
Source: Curr Res Food Sci. 2024 Aug 31;9:100834. doi: 10.1016/j.crfs.2024.100834 (PMC11403449; doi:10.1016/j.crfs.2024.100834)
Supplement: Multimedia component 1 [file mmc1.docx]

Table S1. Adaptation of AV in AR, MR and VR studies.

| **References** | **Main Finding** | **Type(s) of Reality** | **Environment used** | **Food Product/Items** | **Practical Implication** | **Adaptation to Augmented Virtuality** |
| --- | --- | --- | --- | --- | --- | --- |
| (Jeganathan & Szymkowiak, 2023) | The study found that environmental embedding and product liking are mediated by mental imagery quality, affecting the indirect relationship between the two. | AR | - AR environment. - Real-time context for product evaluation. - Embedded product in respondent's local environment | - Various food products | Effective AR product presentation can improve consumer evaluation, resulting in increased product liking, repurchase intentions, and positive word-of-mouth marketing for food retailers. | - Like the study's AR features like simulated physical control and environmental embedding, Augmented Virtuality (AV) could examine how real food products in a virtual environment affect cognitive, affective, and behavioural consumer responses. This adaptation could examine how AV affects food product mental imagery, evaluation, liking, and purchase intention. - Research could examine how embedding 3D models of food products into virtual environments can improve mental imagery generation and transformation, similar to how AR does. - Research on the effects of AV on consumer perceptions and behaviours, including food product evaluation, liking, and purchase intentions, could inform adaptation strategies. |
| (Low et al., 2021) | Researchers found that consumers can be categorised by their emotional response to tea-break snacks, with one emotionally disengaged group and two positively engaged groups at varying intensity and qualities. | MR | - Sensory booths, - Evoked café - Real cafe | - Tea-break snacks | Context is crucial for accurate product response insights from consumer emotional response data.  Mixed-reality methodology may be effective for evaluating consumer responses to ecologically valid food products. Segmenting consumers based on emotional responses can reveal product engagement and preferences. | - The research can be applied to augmented reality (AV) by incorporating real elements or food products into a virtual environment, similar to how mixed-reality was used to simulate a café setting for tea-break snack emotional responses. This adaptation can be useful in real-life settings, such as cafés and food product evaluations, as shown in the study. Consumer emotional response patterns determine whether an immersive mixed-reality context can evoke real-life emotions. To create a meaningful and ecologically valid experience, it is important to consider how consumers emotionally engage with virtual elements or food products when adapting AV. |
| (Fritz et al., 2023) | Research indicates that AR display enhances food item attractiveness and purchase likelihood by boosting mental simulation and personal relevance. | AR | - Real-time environment in field studies and controlled laboratory studies. - University cafe frequented by students and staff in a field experiment. | - Various food products on the presentation of both desirable food items like parmesan fries and less-desirable food items like fermented trout. | Investing in AR product viewing can be beneficial for the food and beverage industry. | - Augmented Virtuality can enhance food attractiveness and purchase likelihood by integrating real elements into virtual environments, making food items more appealing and relevant. |
| (Mishra et al., 2021) | The study found that multimodal technologies, particularly AR, increase visual appeal, emotional appeal, and purchase intentions for hedonic products over utilitarian products. | AR | - Multisensory and haptic interfaces were used in the research study. | - Various food products | The study suggests that online marketers can use AR and VR to improve user experience and influence consumer behaviour. | - Augmented Virtuality (AV) can incorporate real objects or food into a virtual environment, combining AR and VR. - AV adaptation creates a multisensory environment that boosts visual, emotional, and purchase intentions. - AV can create a realistic computer-generated multisensory environment where users can interact with virtual objects in real time, improving the experience. - Integrating real elements into virtual environments can enhance user engagement and adoption, potentially increasing satisfaction. |
| (Korsgaard et al., 2020) | The main finding of the study was that energy intake did not change, but the virtual living room environment was perceived to be more energetic and pleasant, and meals eaten there were rated as higher quality than those eaten in a lab. | MR | - Mixed-reality systems with virtual living room and real lab environment. - Virtual environment for eating, social interactions with avatars. | - Cakes - Boxed juices | Use mixed-reality systems to create virtual dining environments for older adults, potentially increasing food intake and reducing social isolation. | - Augmented Virtuality (AV) can use the research to improve older participants' eating experiences, especially in socially isolated situations. This adaptation can help create a more energetic and pleasant meal experience, leading to higher food quality perception and increased consumption. Like the study's approach of engaging older adults in conversations with remotely located friends, AV systems can prioritise avatar-based social interactions. This adaptation can reduce loneliness and improve mealtime moods, making the experience better. - As shown in the research where the virtual environment improved participants' food experiences, immersive technologies may affect food quality and taste. Explore how environmental factors impact the ecological validity of immersive technologies and meal perception through this adaptation. |
| (Mellos & Probst, 2022) | The research found that nutrition students' food portion estimate skills may improve with AR tools. | AR | - Online, AR, and infographic tools were used for food portion estimation. | - Various food products [Scotch fillet, chicken (diced), muesli (no milk), rice (white, long grain), milk (full fat, cow), cheese (tasty, pre-sliced), and banana (with peel)]. | Augmented reality technology, especially AR tools, can enhance nutrition students' food portion estimation skills. | - Augmented Virtuality (AV) could use real food products in a virtual environment to train food portion estimation skills, similar to how augmented reality (AR) was used in the study. Consider creating a virtual environment for users to interact with real food products and improve their portion estimation skills. |
| (Fuchs et al., 2020) | The study found that MR headset-mediated visual treatments positively impacted beverage and food choices, reducing sugar, energy intake, and saturated fat intake and increasing healthy product selection. | MR | - Real-world retail environment. | - Various food products | The study suggests using MR headsets for real-time interventions to improve food choices and reduce sugar and energy intake. | - Following the research, Augmented Virtuality (AV) can integrate real food products into a virtual environment using MR headset-mounted cameras for computer vision-based diet-related activity detection and real-time interventions to promote healthy food choices. Combining Augmented Reality and Virtual Reality elements can improve user experiences in making healthier food choices. - The adaptation can use automatic CV-based food item identification and real-time visual interventions via MR headsets, which are expected to become popular. This method improves image classification under realistic conditions, enabling the incorporation of real food elements into virtual environments for dietary tracking and interventions. |
| (Torrico, 2021) | The study paper focuses on developing new methodologies to quantify customers' sensory, emotional, and physiological responses to food products. | VR | - Immersive virtual reality and augmented reality environments were utilized. - Home-use tests were conducted to enhance sensory experiences. | - Plant-based yogurt products - Insect-based food snacks | The study suggests using novel sensory science methods to improve food and beverage product evaluation techniques. | - Augmented Virtuality (AV) can be used to study how immersive virtual reality technologies affect food sensory perception, as shown in beef, chocolate, and wine studies. Real food products can be integrated into virtual environments to study consumer perceptions and responses. Researchers can study the impact of context on consumer sensory experiences by incorporating real food products into virtual reality environments, as demonstrated in the wine tasting study. Research suggests that Augmented Virtuality can improve food and beverage product evaluation by using biometric measurements and immersive technologies to measure consumers' sensory, emotional, and physiological responses. |
| (Crofton et al., 2021) | The study found that particular contexts, like a VR restaurant for beef and a countryside for chocolate, significantly affect participants' sensory responses to food products compared to traditional lab settings. | VR | - Traditional sensory booths - VR restaurant - VR Irish countryside - VR busy city | - Beef steaks - Milk chocolate | The study suggests that contextual settings can significantly affect participants' sensory responses to food products compared to traditional sensory lab conditions. | - The research suggests incorporating real food products into virtual environments using Augmented Virtuality (AV), similar to how VR was used to simulate eating environments for beef steaks and chocolate. Using AV technology to create immersive contextual settings can improve sensory perception of food products, aligning with the research goal of examining the impact of environmental contexts on participants' hedonic responses. |
| (Seo, 2020) | Environmental cues around foods and beverages can modulate consumer perception, emotional responses, and behaviour, highlighting the importance of sensory cues from surrounding contexts in understanding food product consumer behaviour. | VR | - Immersive technologies manipulated environmental variables for consumer perception. - Olfactory cues from wait staff influenced patrons' dining experiences. | - Various food products | The research suggests that environmental cues around food and beverages can influence consumer perception and behaviour, requiring consideration by product developers, sensory professionals, retailers, marketers, and business owners. | - Incorporating real food products into virtual environments using immersive technologies such as augmented reality, mixed reality, or simulated immersion can capture contextual influences on consumer perception and behaviour towards food products. AV can focus on creating immersive contexts that match environmental sensory cues to influence consumer perception, liking, and willingness to pay for food and beverage products. - Using AV technology in immersive settings like wineries or bars can increase consumer engagement and highlight the potential of simulated contexts as sensory nudges in real-life settings.Incorporating real food products into virtual environments using immersive technologies such as augmented reality, mixed reality, or simulated immersion can capture contextual influences on consumer perception and behaviour towards food products. AV can focus on creating immersive contexts that match environmental sensory cues to influence consumer perception, liking, and willingness to pay for food and beverage products. - Using AV technology in immersive settings like wineries or bars can increase consumer engagement and highlight the potential of simulated contexts as sensory nudges in real-life settings. |
| (Bhavadharini et al., 2023) | The research paper uses immersive technologies like virtual, augmented, and mixed reality to evaluate food consumer behaviour, focusing on product selection, shopping behaviour, and emotional influence on product choices. | AR, MR, VR | - AR-D view with cows in a dairy farm. - AR-C view with coconut and palm trees. | - Various food products | To analyse consumer behaviour in food selection studies, immersive technologies such as virtual, augmented, and mixed reality can be useful. | - Augmented Virtuality can enhance food consumer behavior studies by creating realistic, interactive virtual environments for better analysis of product selection and emotional responses. |
| (Strecker et al., 2024) | ShoppingCoach, a Diminished Reality (DR) prototype, improves users' compliance with dietary recommendations and speeds up product selection, suggesting that DR could help people eat healthier. | MR | - Simulated shopping scenario with modified product shelf arrangement. - Offline supermarket setting with AR and DR technology integration. | - Crisps - Cereals - Chocolate - Pasta | Consider using Diminished Reality to help users choose healthier products during offline shopping. | - The research paper focuses on Diminished Reality (DR) and its use to promote healthier food choices in offline supermarkets. - The study presents ShoppingCoach, a DR prototype that visually degrades supermarket food products based on their composition deviation from dietary recommendations. Despite not directly addressing Augmented Virtuality (AV), the research paper highlights the use of DR to reduce unhealthy food product visuals and promote healthier alternatives. The study suggests that DR can improve public health by promoting dietary compliance and reducing supermarket decision-making time. |
| (Gere et al., 2021) | The paper introduces current VR knowledge in food science and highlights the most important questions about VR applications in this field. | VR | - Virtual reality environments were used in the food science research. - VR environments included coffeehouses, supermarkets, and eating environments. - VR environments mimicked sensory cues for testing food preferences. | - Various food products | Virtual reality in food science can improve consumer experiences and identify psychological and behavioural factors influencing food choices. | - The research can be used to integrate real food products into virtual environments for sensory testing and consumer experiences. - Create immersive AV environments to test food product consumer behaviour and preferences. - Use touch, taste, and smell in AV environments to improve ecological validity and consumer experience during food testing. - Assess how contextual information in AV environments affects hedonic and emotional reactions to food products, as seen in the study on chocolate acceptability in VR testing conditions. |
| (Zulkarnain, Radványi, et al., 2024) | The study found that combining VR technology with scent identification techniques provides a novel approach to sensory analysis, influencing scent perception and exposure through imagery or the environment. | VR | - Virtual sensory laboratory resembling MATE's sensory booth. | - Scent sticks | The study suggests that integrating VR technology with scent identification methods can improve accuracy and reduce human bias in sensory analysis. | - Augmented Virtuality (AV) can improve sensory testing by integrating real food products into a virtual environment. - As shown in the study, AV can prioritise immersive environments that simulate real-world scenarios with visual and auditory cues to give participants more realistic sensory responses. - AV adaptation can assess scents' effects on cognitive processes in virtual environments, revealing how scents affect sensory perceptions and cognitive resources. |
| (Zulkarnain, Kókai, et al., 2024) | The research found that the VR-enabled virtual sensory booth could improve sensory evaluation and perception studies, particularly in sensory science. | VR | - Virtual reality sensory booth. | - Chocolate biscuits - Orange juices | The research suggests using VR-enhanced virtual sensory booths for sensory evaluation and perception studies in sensory science. | - From the research, Augmented Virtuality (AV) can integrate real food products into a virtual environment to improve sensory evaluation through immersive experiences and interactions. This adaptation can create realistic and engaging environments for sensory analysis and perception studies by combining Augmented Reality and Virtual Reality. - Augmented Virtuality can be used to create a sensory evaluation layer that tests different products using sensory questionnaires like just-about-right (JAR) and check-all-that-apply (CATA). Participants can interact with the virtual sensory booth and provide feedback on their sensory experiences, improving the evaluation process. - The virtual sensory booth can also be configured and calibrated to meet participant needs like height, sample distances, scene clarity, and eyeglasses. Enhancing participant engagement and interaction with the virtual environment through calibration and hand interaction tutorials can improve sensory evaluation effectiveness. |
| (Zulkarnain, Kókai, et al., 2024a) | The main finding of the study was the development of a virtual sensory laboratory with a focus on sensory perception in a virtual environment. | VR | - Virtual reality (VR) environment with sensory booths for bakery items. - Immersive VR technology simulating real-world objects and events. | - 3D Bakery Items - Scent sticks | The practical implications of the study are extensive, benefiting stakeholders such as sensory scientists, the food industry, educators, technology developers, and marketing professionals. | - The research on the virtual sensory laboratory can be adapted to Augmented Virtuality by integrating real-world elements with virtual ones in real time, providing a comprehensive interaction between real and virtual components - Augmented Virtuality can enhance consumer engagement and ecological validity by simulating real contextual environments, similar to the virtual sensory laboratory designed for compatibility with VR gear . - The study's focus on exploring disparities in consumer responses through a virtual environment can be extended to Augmented Virtuality to deepen the understanding of how virtual reality influences consumer behavior in food selection compared to traditional sensory methods |
| (Fuentes et al., 2021) | The research paper highlights the potential for digital technologies to reduce biases and subjectivity in sensory science, incorporating physiological and emotional responses of panellists for accurate data acquisition and interpretation, potentially revolutionising the food and beverage industries. | AR,MR,VR | - Mixed reality, real environment, booths for sensory sessions. - Virtual reality settings for consumer acceptability towards chocolate products. | - Vaious food products | The research can benefit the food and beverage industries by integrating digital technologies in sensory science to improve data acquisition and interpretation. | - This research can be used to integrate real food products into virtual environments using augmented and virtual reality to assess consumer perceptions and preferences. - AV adaptation can enhance consumer responses and acceptability by incorporating real food and beverages into virtual environments to create immersive sensory experiences. |
| (Lombart et al., 2019) | The study found that consumers prefer'moderately' misshapen fruits and vegetables due to their appearance and quality, compared to'slightly' and 'heavily' misshapen ones. | VR | - Virtual supermarket environment. | - Fruits and vegetables | The study suggests that consumers' perceptions and purchasing behaviour of fruits and vegetables are influenced by their appearance abnormality. | - Augmented Virtuality (AV) can adapt from the research by integrating real elements or food products into a virtual environment, like the study's virtual grocery store with fresh fruits and vegetables for participants to interact with using HMD software. - AV adaptation can focus on creating immersive virtual supermarkets that realistically recreate supermarkets, allowing layout and product display changes to improve user experience and engagement. - AV adaptation should evaluate product appearance, quality, and price fairness using interactive virtual prototyping (IVP) to engage users' senses in the virtual environment. - The study suggests that using Head-Mounted Display (HMD) software like Oculus Rift DK2 can enhance users' immersion and experience in the virtual environment, benefiting AV adaptation. |
| (Schouteten et al., 2024) | The study found that food products were more liked when consumed in a congruent VR context, with no difference in elicited emotions. | VR | - Winter and summer VR contexts with natural ambient sounds. | - Watermelon - Cracker - Chocolate truffle | The study suggests that a virtual reality eating environment can significantly affect food product liking, highlighting the potential of VR for sensory research. | - Augmented Virtuality (AV) could incorporate real food products into virtual environments, like the study examined the effects of seasonal food consumption in congruent or incongruent virtual contexts. - AV adaptation could focus on creating immersive virtual environments that mimic natural consumption contexts to improve food product sensory evaluation in a standard setting. - AV adaptation could study immersion by comparing real food products to virtual environments to improve food product acceptance in virtual evaluations. - AV adaptation may use self-report measurements like liking and emotional profiling in a VR context to evaluate the immersiveness and impact of the virtual environment when eating appropriate food products. |
| (Yang et al., 2022) | The study found that the VR session had higher participant engagement than the Evoked session, with audio, VR time, and realistic simulations enhancing immersion. | VR | - VR environment with 360 video, 3D model, and object tracking. - Realistic bar context with sound recordings and picture slideshow. | - Various food products | The study suggests incorporating training to reduce novelty and improve consumer engagement in VR environments. | - Augmented Virtuality can enhance engagement by combining realistic simulations, audio, and object tracking, similar to VR environments. |
| (Ammann et al., 2020) | The study concluded that sensory studies can be successfully transferred to VR environments, resulting in comparable results to real-life settings. | VR | - Virtual reality environment utilized in sensory science studies. - VR setting for food evaluation with modified visual product properties. | - Orange juice - Grape juice - Lemon cake | The research suggests that sensory studies can be applied to virtual reality environments, yielding results similar to real-life settings. | - The research study successfully adapted a sensory science experiment from real life to a virtual environment using Augmented Virtuality (AV). - The study showed that VR can replicate sensory studies in real life. - Augmented Virtuality allowed researchers to change food product colours without changing their composition. - The study highlights the advantages of VR in experimental designs, particularly in sensory science, highlighting the use of AV methodology for consumer behaviour analysis and product design optimisation. |
| (Chai et al., 2022) | The research reveals that AR/MR technology is primarily used in dietary assessment, food nutrition and traceability, sensory science, retail food chain applications, food education and learning, and precision farming. However, limitations and analytical challenges hinder its use in the food sector. | AR,MR | - Virtual park environment with HMD for eating real food. | - Various food products | Augmented/mixed reality technologies can improve food quality monitoring and visualisation in real-time, revolutionising food manufacturing processes. | - Augmented Virtuality (AV) can use the research to integrate real food products into virtual environments to improve the food industry. - The research suggests prioritising Augmented Virtuality applications in dietary assessment, food nutrition and traceability, food sensory science, retail food chain applications, food education and learning, and precision farming. - AV adaptation uses machine learning, computer vision, and artificial intelligence to overcome analytical challenges in recognising food objects in complex environments. - Next steps for AV adaptation include enhancing wireless data transfer and food object recognition to advance its use in the food industry. |
| (Van Der Laan et al., 2022) | The study found no significant preference for perceived healthy or unhealthy food products across prime conditions. | VR | - NeuroShop environment resembled a Dutch supermarket. | - Various food product/items used in the study included essential grocery items such as snacks, breakfast products, dinner products, and drinks. | The study suggests that the NeuroShop virtual reality fMRI paradigm provides a realistic environment for assessing neural responses during food choices, improving understanding of food-related decision-making processes. | - Augmented Virtuality (AV) could incorporate real food products into a virtual environment, like the virtual NeuroShop fMRI paradigm did. Create ecologically valid choice contexts in the lab using AV technology to study real food products in a virtual setting. |
| (Zhang et al., 2022) | The study found that presenting a meat dish in a red container and a vegetable dish in a blue container led to more vegetable dish choices and fewer meat dish choices, highlighting the impact of container colour on food choices. | VR | - Virtual Chinese fast-food restaurant with red and blue containers. | - Meat and Vegetable dishes commonly seen in local Chinese cuisine. | The study suggests that combining red and blue containers can encourage healthier food choices among consumers. | - Following the contexts, the research paper examines how container colour affects virtual restaurant food ratings and choices. The study used red and blue containers to influence food choices. Augmented Virtuality (AV), a hybrid of Augmented Reality and Virtual Reality, is not directly addressed in the research. From the contexts provided, there is no specific information on how Augmented Virtuality might adapt from this research. |
| (Torrico et al., 2021) | The study found that virtual reality (VR) environments did not significantly impact the liking of full-sugar and no-sugar chocolate attributes, but did impact the intensity of sweetness and emotional responses to the products. | VR | - Positive and negative VR environments - Positive VR: 'Autumn in Blue Mountains'; Negative VR: 'The Glass House'. | - Full-sugar and no-sugar (maltitol) chocolates | VR can improve consumer evaluations and help understand contextual effects. | - The research used Virtual Reality (VR) to assess chocolate products' sensory acceptability and emotional responses. Augmented Virtuality (AV) can be used to incorporate real elements or food products into virtual environments. - By integrating real elements into virtual settings, the adaptation can improve consumers' sensory and emotional responses to food products. - Future studies could use Augmented Reality (AR) headsets to allow participants to evaluate food samples in virtual environments, addressing a limitation in the research study. - Augmented Virtuality can be used to study the holistic relationship between hedonic responses, intensities, and contextual effects on consumer perceptions, similar to the multivariate approach used to analyse environmental conditions and chocolate samples. - By using research on how VR environments affect consumer perceptions, Augmented Virtuality can reveal consumer engagement, emotional connections with food products, and how contextual changes affect sensory assessments. |
| (Torrico et al., 2020) | The main finding of the study was that the context (booths, real, or VR) significantly influenced the perception of the wine's floral aroma, while the liking of sensory attributes remained consistent across different environmental conditions. | VR | - Traditional booths - Bright-restaurant - Dark-restaurant - Bright-VR - Dark-VR | - Wines | The practical implication of the study indicates that virtual reality technology can provide a more realistic and engaging environment for sensory evaluation compared to traditional sensory booths. | - The research paper discusses the limitations of virtual reality (VR) in wine tasting evaluations, highlighting the inability to assess the appearance of the sample and the lack of re-tasting of test samples under the VR testing process. - The study suggests that future technologies like augmented reality (AR) could overcome these limitations and provide a more immersive approach than VR, emphasizing the need for future studies in this area. - Augmented Virtuality can be adapted by incorporating AR technology to enhance the immersive experience of wine tasting evaluations, allowing participants to assess the appearance of the sample and potentially enabling re-tasting of test samples, thus improving the overall evaluation process. |

References:

Ammann, J., Stucki, M., & Siegrist, M. (2020). True colours: Advantages and challenges of virtual reality in a sensory science experiment on the influence of colour on flavour identification. *Food Quality and Preference*, *86*, 103998. https://doi.org/10.1016/j.foodqual.2020.103998

Bhavadharini, B., Monica, V., Anbarasan, R., & Mahendran, R. (2023). Virtual, augmented, and mixed reality as a versatile tool in food consumer behavior evaluation: Recent advances in aroma, taste, and texture incorporation. *Comprehensive Reviews in Food Science and Food Safety*, *22*(6), 4925–4956. https://doi.org/10.1111/1541-4337.13248

Chai, J. J. K., O’Sullivan, C., Gowen, A. A., Rooney, B., & Xu, J.-L. (2022). Augmented/mixed reality technologies for food: A review. *Trends in Food Science & Technology*, *124*, 182–194. https://doi.org/10.1016/j.tifs.2022.04.021

Crofton, E., Murray, N., & Botinestean, C. (2021). Exploring the Effects of Immersive Virtual Reality Environments on Sensory Perception of Beef Steaks and Chocolate. *Foods*, *10*(6), 1154. https://doi.org/10.3390/foods10061154

Fritz, W., Hadi, R., & Stephen, A. (2023). From tablet to table: How augmented reality influences food desirability. *Journal of the Academy of Marketing Science*, *51*(3), 503–529. https://doi.org/10.1007/s11747-022-00919-x

Fuchs, K., Haldimann, M., Grundmann, T., & Fleisch, E. (2020). Supporting food choices in the Internet of People: Automatic detection of diet-related activities and display of real-time interventions via mixed reality headsets. *Future Generation Computer Systems*, *113*, 343–362. https://doi.org/10.1016/j.future.2020.07.014

Fuentes, S., Tongson, E., & Gonzalez Viejo, C. (2021). Novel digital technologies implemented in sensory science and consumer perception. *Current Opinion in Food Science*, *41*, 99–106. https://doi.org/10.1016/j.cofs.2021.03.014

Gere, A., Zulkarnain, A. H. B., Szakál, D., Fehér, O., & Kókai, Z. (2021). Virtual reality applications in food science. Current knowledge and prospects. *Progress in Agricultural Engineering Sciences*, *17*(1), 3–14. https://doi.org/10.1556/446.2021.00015

Jeganathan, K., & Szymkowiak, A. (2023). Playing with food – The effects of augmented reality on meal perceptions. *Food Quality and Preference*, *111*, 104969. https://doi.org/10.1016/j.foodqual.2023.104969

Korsgaard, D., Bjorner, T., Bruun-Pedersen, J. R., Sorensen, P. K., & Perez-Cueto, F. J. A. (2020). Eating together while being apart: A pilot study on the effects of mixed-reality conversations and virtual environments on older eaters’ solitary meal experience and food intake. *2020 IEEE Conference on Virtual Reality and 3D User Interfaces Abstracts and Workshops (VRW)*, 365–370. https://doi.org/10.1109/VRW50115.2020.00079

Lombart, C., Millan, E., Normand, J.-M., Verhulst, A., Labbé-Pinlon, B., & Moreau, G. (2019). Consumer perceptions and purchase behavior toward imperfect fruits and vegetables in an immersive virtual reality grocery store. *Journal of Retailing and Consumer Services*, *48*, 28–40. https://doi.org/10.1016/j.jretconser.2019.01.010

Low, J. Y. Q., Lin, V. H. F., Jun Yeon, L., & Hort, J. (2021). Considering the application of a mixed reality context and consumer segmentation when evaluating emotional response to tea break snacks. *Food Quality and Preference*, *88*, 104113. https://doi.org/10.1016/j.foodqual.2020.104113

Mellos, I., & Probst, Y. (2022). Evaluating augmented reality for ‘real life’ teaching of food portion concepts. *Journal of Human Nutrition and Dietetics*, *35*(6), 1245–1254. https://doi.org/10.1111/jhn.13016

Mishra, A., Shukla, A., Rana, N. P., & Dwivedi, Y. K. (2021). From “touch” to a “multisensory” experience: The impact of technology interface and product type on consumer responses. *Psychology & Marketing*, *38*(3), 385–396. https://doi.org/10.1002/mar.21436

Schouteten, J. J., Van Severen, A., Dull, D., De Steur, H., & Danner, L. (2024). Congruency of an eating environment influences product liking: A virtual reality study. *Food Quality and Preference*, *113*, 105066. https://doi.org/10.1016/j.foodqual.2023.105066

Seo, H.-S. (2020). Sensory Nudges: The Influences of Environmental Contexts on Consumers’ Sensory Perception, Emotional Responses, and Behaviors toward Foods and Beverages. *Foods*, *9*(4), 509. https://doi.org/10.3390/foods9040509

Strecker, J., Wu, J., Bektaş, K., Vaslin, C., & Mayer, S. (2024). ShoppingCoach: Using Diminished Reality to Prevent Unhealthy Food Choices in an Offline Supermarket Scenario. *Extended Abstracts of the CHI Conference on Human Factors in Computing Systems*, 1–8. https://doi.org/10.1145/3613905.3650795

Torrico, D. D. (2021). Novel Techniques to Measure the Sensory, Emotional, and Physiological Responses of Consumers toward Foods. *Foods*, *10*(11), 2620. https://doi.org/10.3390/foods10112620

Torrico, D. D., Han, Y., Sharma, C., Fuentes, S., Gonzalez Viejo, C., & Dunshea, F. R. (2020). Effects of Context and Virtual Reality Environments on the Wine Tasting Experience, Acceptability, and Emotional Responses of Consumers. *Foods*, *9*(2), 191. https://doi.org/10.3390/foods9020191

Torrico, D. D., Sharma, C., Dong, W., Fuentes, S., Gonzalez Viejo, C., & Dunshea, F. R. (2021). Virtual reality environments on the sensory acceptability and emotional responses of no- and full-sugar chocolate. *LWT*, *137*, 110383. https://doi.org/10.1016/j.lwt.2020.110383

Van Der Laan, L. N., Papies, E. K., Ly, A., & Smeets, P. A. M. (2022). Examining the neural correlates of goal priming with the NeuroShop, a novel virtual reality fMRI paradigm. *Appetite*, *170*, 105901. https://doi.org/10.1016/j.appet.2021.105901

Yang, Q., Nijman, M., Flintham, M., Tennent, P., Hidrio, C., & Ford, R. (2022). Improving simulated consumption context with virtual Reality: A focus on participant experience. *Food Quality and Preference*, *98*, 104531. https://doi.org/10.1016/j.foodqual.2022.104531

Zhang, W., Wang, C., & Wan, X. (2022). Influence of container color on food ratings and choices: Evidence from a desktop VR study. *Food Quality and Preference*, *96*, 104448. https://doi.org/10.1016/j.foodqual.2021.104448

Zulkarnain, A. H. B., Kókai, Z., & Gere, A. (2024a). Assessment of a virtual sensory laboratory for consumer sensory evaluations. *Heliyon*, *10*(3), e25498. https://doi.org/10.1016/j.heliyon.2024.e25498

Zulkarnain, A. H. B., Kókai, Z., & Gere, A. (2024b). Immersive sensory evaluation: Practical use of virtual reality sensory booth. *MethodsX*, *12*, 102631. https://doi.org/10.1016/j.mex.2024.102631

Zulkarnain, A. H. B., Radványi, D., Szakál, D., Kókai, Z., & Gere, A. (2024). Unveiling aromas: Virtual reality and scent identification for sensory analysis. *Current Research in Food Science*, 100698. https://doi.org/10.1016/j.crfs.2024.100698
